# Right main bronchus rupture associated with blunt chest trauma: a case report

**DOI:** 10.1186/s12245-019-0258-3

**Published:** 2019-12-10

**Authors:** Carime Díaz, Daniel F. Carvajal, Eliana I. Morales, Saveria Sangiovanni, Liliana Fernández-Trujillo

**Affiliations:** 1 Department of Emergency Medicine, Fundación Valle del Lili, Cali, Colombia; 2 Faculty of Health Sciences, Universidad Icesi, Cali, Colombia; 3 Department of Internal Medicine, Pulmonology Service, Fundación Valle del Lili, Cali, Colombia; 4 Clinical Research Center, Fundación Valle del Lili, Cali, Colombia; 5 Department of Internal Medicine, Pulmonology Service, Interventional Pulmonology, Fundación Valle del Lili, Avenida Simón Bolívar. Cra. 98 No. 18-49, Tower 6, 4th Floor, Cali, 760032 Colombia

**Keywords:** Chest injuries, Lung injuries, Early diagnosis, Bronchoscopy, Conservative treatment

## Abstract

**Background:**

Tracheobronchial injury is one of the least common injuries in the scenario of blunt chest trauma. However, around 81% of patients with airway injury die immediately or before arriving at the emergency department due to tension pneumothorax. It presents with non-specific signs and symptoms challenging prompt diagnosis.

**Case presentation:**

A 15-year-old adolescent who was riding a bicycle suffered an accident when he fell down a cliff, approximately 5 m deep. Upon admission to the emergency department, he presented with signs of respiratory distress. The airway was secured and a thoracoabdominal angiography was performed. The image reported pneumomediastinum, a small right pneumothorax, areas of pulmonary contusion, and an image of loss of continuity in the anterior superior wall of the right main bronchus highly suggestive of bronchial rupture. The bronchial lesion was then confirmed by fiberoptic bronchoscopy. Taking into account the patient’s characteristics, conservative management was chosen, and the patient was transferred to the intensive care unit (ICU) where protective tracheal intubation was performed.

**Conclusions:**

A delay in diagnosis increases the rate of complications, mainly infectious complications and the formation of granulation tissue that could potentially obstruct the airway, impacting the patient’s outcome. The first step in the management of these patients is securing the airway, which should be done immediately. The gold standard for the diagnosis and characterization of airway injuries is bronchoscopy as it is the most effective tool to assess topography, extent, and depth of the lesion.

## Background

Tracheobronchial injury is one of the least common injuries in the scenario of blunt chest trauma. Nevertheless, it has a high mortality rate [1, 2]; tears or ruptures of the airway are seen in 0.5 to 1.5% of all cases [3, 4] and around 81% of patients with airway injury die immediately or before arriving at the emergency department due to tension pneumothorax [5, 6]. It presents with non-specific signs and symptoms challenging prompt diagnosis. In general, the most common symptom is dyspnea and the main findings on chest X-ray (CXR) are pneumothorax, pneumomediastinum, subcutaneous emphysema, and atelectasis [7].

A delay in diagnosis increases the rate of complications, mainly infectious complications (empyema, hilar abscesses, mediastinitis, pneumonia, etc.) and the formation of granulation tissue that could potentially obstruct the airway [2], impacting the patient’s outcome. We present a case report of a patient who suffers right main bronchus rupture after blunt chest trauma in the setting of a high-impact mechanism, emphasizing on the importance of early diagnosis and treatment to avoid major complications.

## Case presentation

**Fig. 1:**
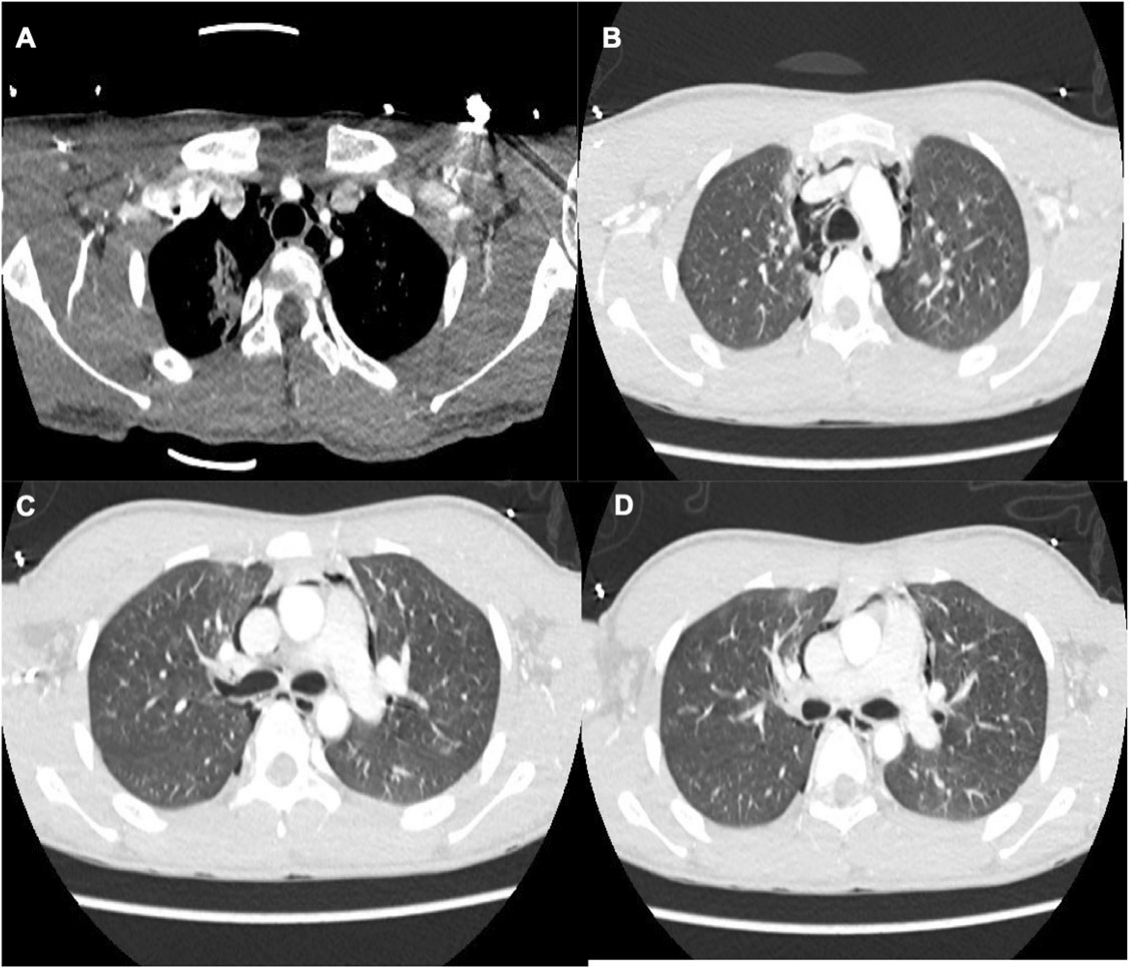
CT angiography. **a** Pulmonary contusion in the apical portion of the right lung and mediastinal emphysema. **b**, **c**, **d** Mediastinal emphysema dissecting central structures

**Fig. 2:**
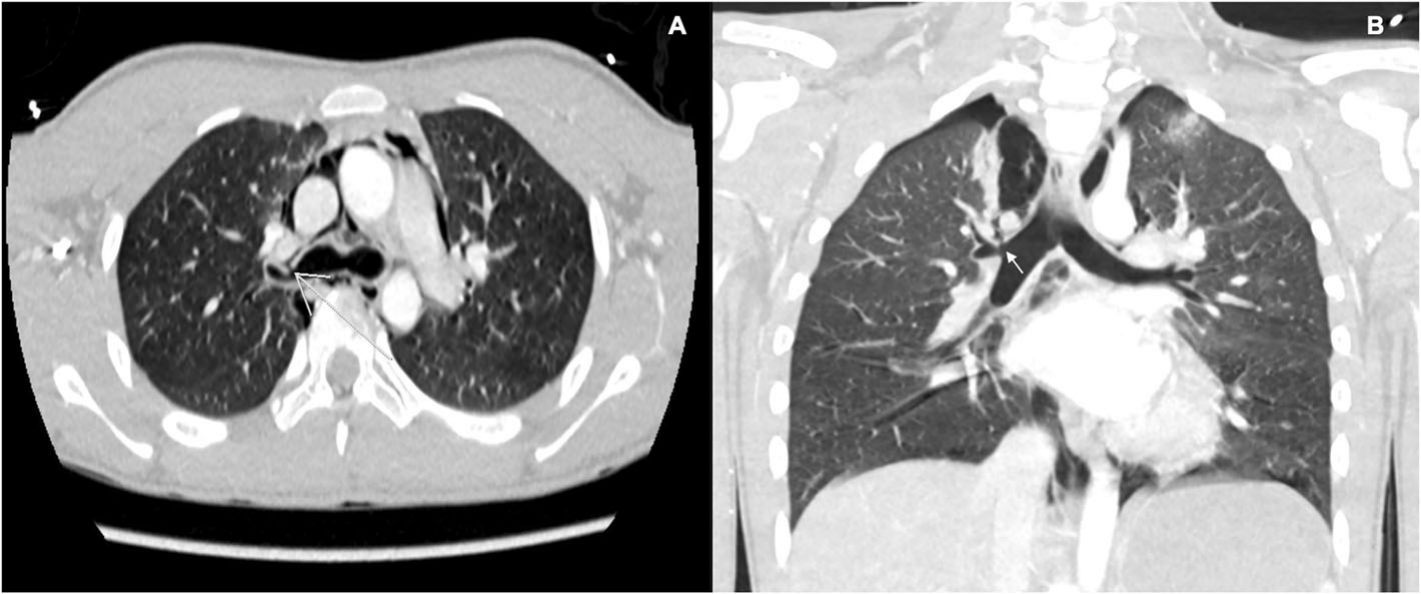
Computed tomography angiography. **a** Transverse section. Right main bronchus rupture and mediastinal emphysema. **b** Sagittal section. Right main bronchus rupture, mediastinal emphysema, and right pneumothorax

A 15-year-old adolescent who was riding a bicycle suffered an accident when he fell down a cliff, approximately 5 m deep. Upon admission to the emergency department, he presented with a moderate traumatic brain injury with a Glasgow Coma Scale (GCS) score of 9/15, stigmas of trauma in the anterior chest wall, and signs of respiratory distress. The airway was secured and a thoracoabdominal computed tomography angiography was performed, as part of trauma work-up; the image reported pneumomediastinum, a small right pneumothorax, areas of pulmonary contusion, and an image of loss of continuity in the anterior superior wall of the right main bronchus highly suggestive of bronchial rupture. The bronchial lesion was then confirmed by fiberoptic bronchoscopy, in which a linear 1-cm rupture at the level of the root of the right main bronchus, in its lateral aspect, with fracture of the entire bronchial wall was seen. There was no air leakage or bleeding. The left bronchial tree was normal. Given these findings, conservative management was chosen, and the patient was transferred to the intensive care unit (ICU) where protective tracheal intubation was performed. The ventilator settings during the patient’s ICU stay were respiratory rate of 12 breaths per minute, tidal volume of 6–8 ml/kg, I/E 1:3, PEEP 5 cm H_2_O, and plateau pressure of < 30 mmHg. Forty-eight hours later, there was a spontaneous resolution of the pneumothorax and pneumomediastinum, with limited subcutaneous emphysema. On day 4, the patient was successfully extubated and strict follow-up was performed, without evidence of swallowing or respiratory disorders, which allowed for the patient’s discharge 1 week later (Figs. 1 and 2).

## Discussion and conclusions

As with all trauma patients, initial management includes rapid physical examination followed by control of bleeding and shock, with special attention to stabilizing the airway [5]. Airway injury presents with non-specific signs and symptoms, thus making diagnostic evaluation misleading and requiring a high index of suspicion to make an accurate diagnosis [2]. It is important to highlight that these patients tend to have clavicle and rib fractures and that the right main bronchus is most commonly affected due to its anatomy [8]. The most common symptoms are dyspnea and respiratory distress which have been reported in 76 to 100% of patients [4]. Sternal tenderness, hemoptysis, hoarseness, and Hamman’s sign have also been described [2].

The main findings on chest X-ray (CXR) are subcutaneous emphysema (35–85%), pneumomediastinum (60%), and pneumothorax (20–50%). Furthermore, when there is complete transection of a main bronchus, atelectasis and an “absent hilum” can be seen [4]. In the scenario of a patient with pneumothorax secondary to blunt chest trauma, which does not improve despite tube thoracostomy, a bronchial rupture should be suspected [3, 5, 6]. The pneumothorax is caused by the communication of the bronchial lesion with the pleural space, while the pneumomediastinum and subcutaneous emphysema occur when there is a lack of communication between the lesion and pleural space.

In order to diagnose airway injury, suspension of the lesion is key. After a CXR is obtained in the emergency department, contrasted three-dimensional images are usually performed as part of the work-up of trauma patients and their findings can be very useful. However, its use for diagnosing distal airway injuries is still controverted, probably because it cannot be done in all patients, specifically in hemodynamically unstable patients or those with an unstable airway [4]. In a study by Scaglione et al., computed tomography angiography (CTA) of the chest captured an airway injury in 10 of 14 patients (71%) with bronchial rupture [1]. However, the absence of a lesion in a CTA is not enough to rule out a tracheobronchial injury [4].

The gold standard for the diagnosis and characterization of airway injuries is bronchoscopy, with evaluation of both the airway and digestive tract as appropriate, being the most effective tool to assess topography, extent, and depth of the lesion [2, 3, 5, 9, 10]. When the diagnosis is delayed, patients present with progressive dyspnea, stridor, and complete atelectasis of the distal lung [6], probably secondary to granulation tissue, which forms between 1 and 4 weeks after the event [4].

The first step in the management of these patients is securing the airway, which should be done immediately. It is recommended to perform intubation by bronchoscopy with a conscious patient, thus avoiding induction and neuromuscular blockade which is contraindicated in patients with an unstable airway and in those who require multiple assessments and hemodynamic stabilization [4, 5, 8].

Regarding surgical treatment, it is appropriate for lesions larger than 2 cm, although some authors tolerate up to 4 cm. Mainly it is based on debridement of the lesion and primary repair [5]. Conservative management is only chosen for small lesions, with no evidence of progression of subcutaneous emphysema and that have resolution of the pneumothorax and no evidence of infection. Conservative treatment focuses on effective mechanical ventilation with positive pressure at the end of expiration (PEEP) and low tidal volume [6, 8, 10]. On the other hand, mucosal defects can heal with conservative management, but the result may be uncertain, since the lesion might develop granulation tissue and airway stenosis may occur, requiring subsequent interventions [6]. Such patients should remain under surveillance in the ICU and be taken to surgery in case of imminent loss of airway permeability or associated complications [6, 10].

In all patients who suffer blunt chest trauma in the setting of a high energy mechanism, airway injuries should always be suspected. The diagnostic algorithm includes physical examination, CXR, CT scans, and confirmation by bronchoscopy, which is the gold standard. An opportune diagnosis impacts on the development of potentially fatal complications and overall mortality. Not all lesions require surgical management, with conservative management being the optimal therapeutic alternative in moderate cases. A strict surveillance and consequent ventilatory goals guarantee that conservative management has a good prognosis, avoiding highly invasive interventions that are not without risk, as in the case presented.

## Data Availability

All data and material are available for sharing if needed.

## Abbreviations

CTA: Computed tomography angiography
CXR: Chest X-ray
GCS: Glasgow Coma Scale
ICU: Intensive care unit
PEEP: Positive pressure at the end of expiration
